# Trends and Patterns of Urodynamic Studies in U.S. Males, 2000–2012

**DOI:** 10.1371/journal.pone.0133657

**Published:** 2015-07-21

**Authors:** Mitchell M Conover, Michele Jonsson Funk, Alan C. Kinlaw, Kristy M. Borawski, Jennifer M. Wu

**Affiliations:** 1 Department of Epidemiology, Gillings School of Global Public Health, University of North Carolina at Chapel Hill, Chapel Hill, North Carolina, United States of America; 2 Department of Urology, University of North Carolina at Chapel Hill, Chapel Hill, North Carolina, United States of America; 3 Department of Obstetrics and Gynecology, University of North Carolina at Chapel Hill, Chapel Hill, North Carolina, United States of America; Baylor College of Medicine, UNITED STATES

## Abstract

**Objective:**

To evaluate trends in urodynamic procedures in the U.S. males from 2000–2012 and determine if a 2010 decline in reimbursement was associated with decreased utilization.

**Subjects and methods:**

We analyzed 2000–2012 administrative healthcare claims from Truven Health’s Marketscan Database and evaluated males ≥18 years of age. We identified cystometrograms and any concurrent procedures using procedure billing codes. Covariates included age, year of cystometrogram, region and associated diagnosis codes. We estimated standardized cystometrogram utilization rates per 10,000 person-years (PY). We used age, region, and calendar year adjusted Poisson regression models to estimate the independent effect of calendar year and region.

**Results:**

During 127,558,186 PY of observation, we identified 153,168 cystometrograms for an overall utilization rate of 12.0 per 10,000 PY (95% CI 11.9–12.1). Cystometrogram utilization increased with age, peaking at age 85 with a rate of 77.7 per 10,000 PY (95% CI 74.7–80.7). Adjusted cystometrogram utilization rate ratios show that compared to a referent of 2000–2004, utilization was significantly higher in each year 2005 to 2011 among all patients and in 2012 among patients ≥ 65. Standardized utilization rates peaked in 2008 at 12.4 per 10,000 PY (95% CI 12.2–12.6), remained elevated until 2010, then decreased slightly in 2011 and substantially in 2012 to 8.5 per 10,000 PY (95% CI 8.4–8.7).

**Conclusions:**

Utilization of urodynamic procedures increased until 2010 and decreased thereafter. Utilization was greatest among men older than 65.

## Introduction

Urodynamic studies (UDS) are used to provide functional information regarding bladder storage and emptying in men with lower urinary tract symptoms (LUTS). [1,2] While invasive UDS may assist with the evaluation of LUTS, these costly procedures are associated with a risk of infection, urethral trauma and pain. [3–5] Despite the widespread use of UDS in the United States, limited population-based data exist regarding recent patterns and trends of these procedures among adult males.

To date, only two prior studies have evaluated UDS rates that included males, one of which was only published in abstract form (Mueller E, Kenton K. Urodynamic procedures are on the rise in the United States [abstract]. J Urol 2009;181:867–8), but neither study provided male-specific rates or considered data past 2007. [6] It is critically important to assess current trends, as a major factor that may have impacted UDS rates is a change in Current Procedural Terminology (CPT) coding and the associated decrease in reimbursement that occurred in 2010. [7–10] Anecdotally, some believe that the decrease in reimbursement may have decreased rates of UDS; however, no data exist to support, or to refute, this claim. More importantly, rates of UDS should not change based on reimbursement but instead on practice guidelines or changes in the evidence base regarding UDS.

We were interested in testing the hypothesis that changes in UDS CPT coding and reimbursement were associated with a decrease in UDS utilization after 2010. Thus, the objective of this study was to evaluate trends in UDS utilization rates from 2000–2012 in adult U.S. males. We also sought to address gaps in the existing literature by providing current UDS utilization rates since 2007 in a large population-based cohort.

## Materials and Methods

We evaluated outpatient administrative health claims from the MarketScan database (Copyright 2013 Truven Health Analytics Inc.; all rights reserved) from 2000–2012 which includes de-identified, adjudicated, healthcare claims from approximately 150 payers in the U.S from employer-based plans. The database includes the MarketScan Commercial Claims & Encounters data for individuals < 65 years of age, and the MarketScan Medicare Supplemental data, which includes healthcare claims for employer-supplemented Medicare plans for individuals ≥ 65. [11,12] These data have been validated by Truven Health Analytics to ensure completeness, accuracy, and reliability. [13] This database includes a significant proportion of those with employer-based insurance–e.g. in 2011, this database included 53 million of the approximately 170.1 million individuals in the U.S. with employment-based insurance. [14] The authors cannot fully disclose study data due to the sensitive health information available in administrative claims. However, any researcher can access MarketScan data (http://truvenhealth.com/your-healthcare-focus/life-sciences/data-databases-and-online-tools; http://truvenhealth.com/Portals/0/Users/031/31/31/PH_13434%200314_MarketScan_WP_web.pdf) by purchasing a data license from Truven Health Analytics (http://truvenhealth.com/).

From a study population including all adult (18 years or older) men, we identified index CMGs using Current Procedural Terminology (CPT) codes [51725, 51726, 51727, 51728, 51729]. We considered CMGs the primary procedure for complex UDS, and then identified claims for concomitant UDS performed on the same date as the index CMG, including urethral pressure profiles (UPP) [51772, 51727, 51729], voiding pressure studies (VP) [51795, 51797, 51728, 51729], electromyography studies (EMG) [51784, 51785], fluoroscopy/video-urodynamics [74430, 74450, 74455, 76000, 76001], and uroflowmetry (UFR) [51741].

We estimated rates of CMG procedures stratified by age, calendar year, and region of service (i.e. Northeast, North Central, South, West, unknown) by dividing the total frequency of CMGs (allowing subjects to contribute multiple CMGs) by the total person-time in each stratum. Stratifying estimates by calendar year and age simultaneously allowed us to evaluate temporal trends in utilization, while accounting for changing age profiles over time. To obtain the person-time denominators for rate calculations, we aggregated all of the person-time (i.e. time spent enrolled in insurance plans captured by MarketScan) accrued in the database, during which men were assumed to be continuously eligible for UDS.

To evaluate trends in utilization rates by region and calendar year that were not confounded by changing regional and age distributions in the database over time, we calculated standardized utilization rates and 95% confidence intervals (CI). [15] We uniformly reweighted stratum-specific data from our database to resemble a standard population of U.S. males with employer-provided health plans (below age 65) or with employer-supplemented Medicare plans (over age 65), as estimated by the 2010 Current Population Survey. [16] We estimated calendar year utilization rates, standardized by age and region as well as regional utilization rates, standardized by age and calendar year.

We used Poisson regression to estimate utilization rate ratios and 95% CIs by calendar year and region. To evaluate the significance of comparisons we used Wald chi-square statistics. We compared utilization in the three years before and after the 2010 coding change. For a more detailed view of temporal trends in utilization surrounding the coding change, we compared each individual year from 2005–2012 to a baseline period of 2000–2004 and stratified estimates by those over and under age 65. We grouped the first five years of data since 1) they are distal from the (of-interest) 2010 coding change and 2) sparse data in the early years of the database make year-specific estimates less stable.

In 2010, new CPT codes were introduced which bundled payments for urodynamic procedures frequently performed together, leading to an overall decrease in reimbursement for UDS. [7–10] We assessed two common urodynamic procedure bundles (CMG+VP and CMG+VP+UPP), which after 2010 were billed using a single code. Comparing the 2009 [7] and 2010 [9] Physician Fee Schedules, reimbursement rates declined 34.9% for CMG+VP bundles and 48.2% CMG+VP+UPP bundles. To evaluate whether reimbursement decreased in our study population, we measured the median and interquartile range (IQR) of the total payment for CMG+VP bundles and CMG+VP+UPP bundles in the three years leading up to the 2010 coding change (2007–2009) and the three years after (2010–2012).

To further inform our understanding of trends in UDS utilization, we assessed clinical variables, including provider specialty, and diagnoses most associated with the index CMG procedure, identified using diagnosis codes from the International Classification of Diseases, Ninth Revision, Clinical Modification (ICD-9-CM). We assessed the diagnoses associated with CMGs by inspecting all ICD-9 codes recorded on the index CMG’s medical claims or any other concomitant UDS procedures. We aggregated the 30 most frequent diagnosis codes into 15 exclusive categories. We identified provider type for each CMG based on the specialty of the billing physician.

We also calculated the proportion of CMGs performed with VPs, UPPs, EMGs, flouroscopy, and UFR. We defined concomitant procedures as those performed on the same date of service as the index CMG. In order to describe temporal changes in the type/complexity of UDS studies being used, we evaluated these estimated proportions over calendar time. All analyses were conducted with SAS 9.3 (SAS Institute, Cary, NC).

### Ethics Statement

This study was reviewed by University of North Carolina’s institutional review board (study #: 10–0153) and found to be exempt.

## Results

We identified 153,168 CMGs, during 127,558,186 person-years (PY) of observation, (among 126,866 unique patients). Truven Health Analytics increased the number of contributing plans during the study period and, as a result, the size of the study cohort increased substantially over time, accruing 1,371,174 PY in 2000 and 17,974,778 PY in 2012 (Table 1). Thus, the overall crude utilization rate of 12.0 per 10,000 PY (95% CI 11.9–12.1) was weighted more heavily towards the rates in more recent years. Table 1 displays CMG frequencies and person-time data within categories of age, calendar year, and region-of-service.

**Table 1:** Observed person-time and cystometrogram (CMG) procedure frequencies by age, calendar year, and region of service, for U.S. males, 2000–2012.

	Person-years	n
**Age**		
18–24	15,403,276	2,119
25–29	9,147,734	1,587
30–34	10,702,790	2,212
35–39	11,980,431	2,965
40–44	13,194,482	4,616
45–49	14,165,315	7,227
50–54	14,250,656	11,355
55–59	13,212,680	17,532
60–64	10,911,664	23,335
65–69	4,555,209	16,471
70–74	3,605,024	18,787
75–79	2,929,878	19,762
80–84	2,059,548	15,366
85+	1,439,499	9,834
**Year**		
2000	1,371,174	1,493
2001	2,256,601	2,772
2002	3,882,524	4,293
2003	5,962,374	6,112
2004	7,781,069	8,441
2005	8,616,390	10,313
2006	9,476,184	11,580
2007	9,521,170	11,856
2008	13,898,154	19,512
2009	13,781,311	19,486
2010	15,262,824	19,815
2011	17,773,633	21,693
2012	17,974,778	15,802
**Region**		
Northeast	17,855,298	35,312
North Central	32,972,415	47,995
South	50,486,448	48,519
West	24,711,993	19,486
Unknown	1,532,032	1,856

**Fig 1:**
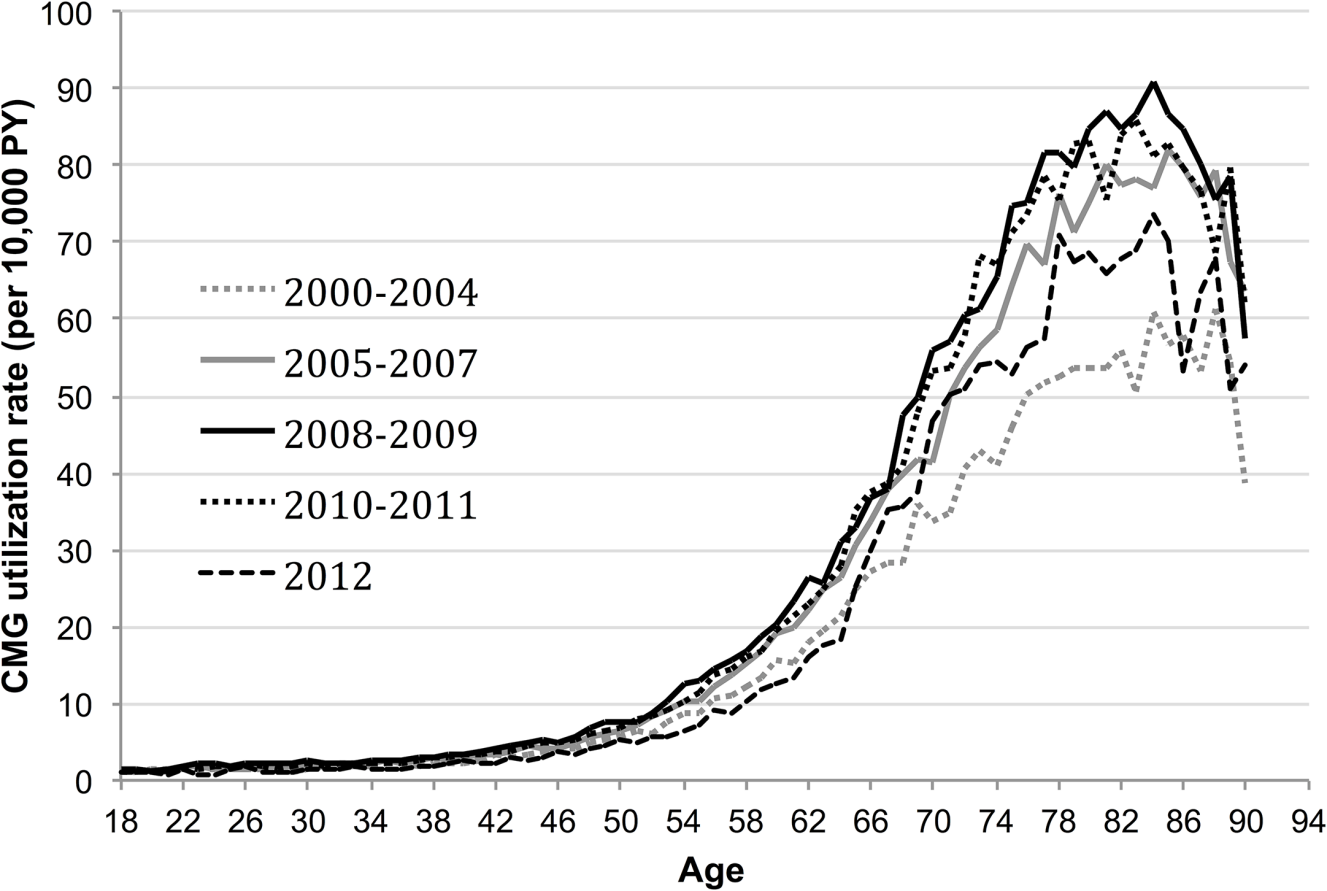
Cystometrogram (CMG) utilization rates per 10,000 person-years by calendar year, U.S. males 2000–2012.

Crude CMG utilization increased with age and peaked at age 84 with a rate of 77.7 (95% CI 74.7–80.8) per 10,000 PY, which was then followed by a decline in utilization. The age-specific utilization rate remained below 10 CMGs per 10,000 PY until age 54. Age-specific CMG utilization rates for different calendar year periods are displayed in Fig 1. The standardized CMG utilization rate increased during the study period, peaking from 2008–2010, then decreased moderately in 2011 and substantially in 2012 (Table 2). The rate was highest in 2008 (12.4 per 10,000 PY, 95% CI 12.2–12.6) and fell to 8.5 per 10,000 PY (95% CI 8.4–8.7) by 2012. The standardized CMG utilization rate estimates also showed wide regional variation. The rate in the Northeast (16.0 per 10,000 PY, 95% CI 15.8–16.3) was substantially higher than the other regions. Utilization was lowest in the Western (7.0 per 10,000 PY, 95% CI 6.8–7.2) region of the U.S. (Table 2).

**Table 2:** Standardized cystometrogram (CMG) utilization rates and adjusted utilization rate ratios by calendar year and region, for U.S. males 2000–2012.

	Utilization rates^a^		Utilization rate ratios^b^			
	per 10k person-years		< 65 years old		≥ 65 years old	
	Rate	95% CI	Rate Ratio	95% CI	Rate Ratio	95% CI
**Calendar Year**						
2000	10.7	(10.0, 11.4)				
2001	9.1	(8.7, 9.5)				
2002	9.0	(8.7, 9.3)	1.00	–	1.00	–
2003	9.0	(8.7, 9.3)				
2004	9.7	(9.5, 10.0)				
2005	10.5	(10.3, 10.8)	1.14	(1.09, 1.19)	1.22	(1.17, 1.28)
2006	11.6	(11.3, 11.8)	1.23	(1.18, 1.28)	1.42	(1.36, 1.49)
2007	11.4	(11.1, 11.6)	1.20	(1.15, 1.25)	1.48	(1.41, 1.54)
2008	12.4	(12.2, 12.6)	1.28	(1.23, 1.32)	1.44	(1.38, 1.49)
2009	12.4	(12.2, 12.6)	1.29	(1.25, 1.34)	1.43	(1.38, 1.49)
2010	12.4	(12.2, 12.6)	1.25	(1.20, 1.29)	1.47	(1.41, 1.52)
2011	11.2	(11.0, 11.3)	1.07	(1.04, 1.11)	1.38	(1.33, 1.43)
2012	8.5	(8.4, 8.7)	0.76	(0.73, 0.79)	1.17	(1.12, 1.21)
**Region**						
Northeast	16.0	(15.8, 16.3)	1.00	–	1.00	–
North Central	11.9	(11.7, 12.0)	0.55	(0.54, 0.56)	0.80	(0.78, 0.83)
South	9.5	(9.3, 9.6)	0.51	(0.50, 0.52)	0.62	(0.60, 0.64)
West	7.0	(6.8, 7.2)	0.33	(0.32, 0.34)	0.45	(0.43, 0.46)
Unknown	–	–	0.69	(0.63, 0.76)	0.62	(0.57, 0.68)

^a^ We estimated standardized utilization rates by year and region by reweighting the age distribution in the data to resemble a standard population of U.S. males with employer-provided health plans (under age 65) and U.S. males with employer-supplemented Medicare plans (above age 65), as estimated by the 2010 Current Population Survey. Calendar year rates are standardized by age and region while regional rates are standardized by age and calendar year (weighting each year of data equally).

^b^ Within each stratum of age (< 65 and ≥ 65), we estimated utilization rate ratios by calendar year using age and region adjusted Poisson models, and by region using age and calendar year adjusted models. We used a referent of 2000–2004 for rate ratios by calendar year and the Northeast for rate ratios by region.

We used Poisson models to estimate adjusted CMG utilization rate ratios. Compared to a baseline period of 2000–2004, the utilization rate was significantly higher in each year 2005–2011 among all patients and in 2012 among patients 65 years and older. For patients younger than 65, the utilization rate in 2012 was significantly lower than the period 2000–2004. Regional variation in utilization of UDS is more pronounced among patients under age 65. Utilization was lowest in the Western region and highest in the Northeast among both patients over and under age 65. Utilization rate ratios obtained using the Poisson model are presented in Table 2 along with 95% confidence intervals.

We observed a decline in reimbursement for urodynamic procedures in our study population after the CPT coding change in 2010. The median total payment for CMG+VP+UPP bundles was $718.75 (IQR: $578.63–$940.79) in the period preceding the coding change (2007–2009), which decreased 38.1% in the period after the coding change (2010–2012) to $444.75 (IQR: $300.58–$641.92). Median total payments for CMG+VP bundles decreased by 29.1%, with a median of $575.00 (IQR: $435.75–$795.31) before the coding change and $407.75 (IQR: $291.79–$567.43) after the coding change. Using adjusted Poisson models to compare utilization in these two periods, we estimated a CMG utilization rate ratio of 0.86 (95% CI 0.84–0.88, p<0.0001), indicating decreased utilization in the period after the 2010 coding change.

**Fig 2:**
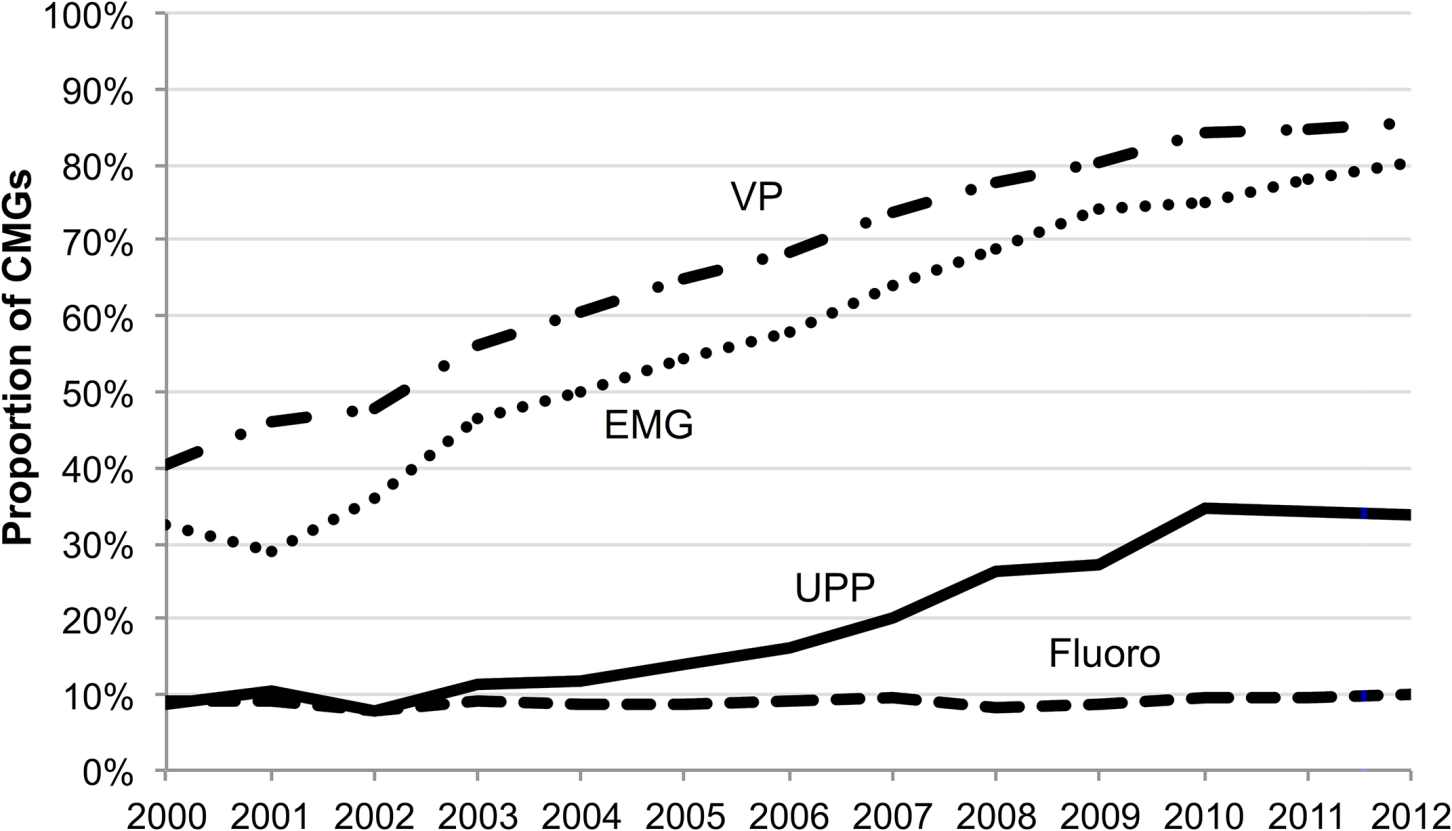
Proportion of cystometrograms (CMG) accompanied by voiding pressure (VP) studies, urethral pressure profiles (UPP), electromyography (EMG), and fluoroscopy procedures by calendar year, U.S. males 2000–2012.

Among patients who had a CMG, UFR occurred concomitantly with 67.1% and VPs with 75.3%, while UPPs, EMGs, and fluoroscopy accompanied 24.9%, 66.7% and 9.1%, respectively. The proportion of CMGs accompanied by EMGs increased monotonically over the study period, while the proportion accompanied by VPs and UPPs flattened out after 2010 (Fig 2). The proportion accompanied by fluoroscopy procedures remained relatively stable just below 10%.

The most frequent diagnoses accompanying CMGs were incomplete emptying/obstruction/retention, frequency/urgency/nocturia, benign prostatic hyperplasia, urge/unspecified urinary incontinence, and neurogenic bladder, which accounted for 31.1%, 25.8%, 20.5%, 12.7%, 8.2% of CMGs ordered, respectively. The majority (70.9%) of CMGs performed on male patients were billed by urologists.

## Discussion

Utilization of CMGs varied widely during the study period. Rates increased from 2000 until 2010, then decreased slightly in 2011 and substantially in 2012. In our study population, which includes men with employer-based private insurance, the introduction of new codes for bundled urodynamic procedures in 2010 led to a substantial decline in reimbursement. This was associated with a decline in utilization of CMGs after the coding change. By 2012, utilization among patients younger than 65 declined to levels below those observed in the period 2000–2004.

Our results show similar trends in UDS rates through 2007 when compared to two prior studies. (Mueller & Kenton [abstract], 2009) [6] The first study analyzed national trends in American Board of Urology certification/recertification applications from 2003 and 2007, and showed a dramatic increase in the number of complex UDS being performed per urologist (Mueller & Kenton [abstract], 2009). However, this study did not provide *rates* of UDS or any male-specific data. Reynolds et al. used administrative claims data from 2002–2007 and evaluated 16,574 CMGs to assess factors associated with complexity of UDS but did not assess temporal trends. [6]

Changing practice patterns may be driven by changes in reimbursement, however other explanations include the publication of new clinical guidelines and evidence on use of urodynamics. Notably, the American Urological Association (AUA) released updated benign prostatic hyperplasia guidelines in 2010, which affirmed that complex CMGs are not indicated in the evaluation of the routine patient with lower urinary tract symptoms, especially one presenting with obstructive symptoms and a urinary flow < 10mL/sec. [4] UDS may be helpful in cases where Qmax is >10mL/sec or confounding disease states exist. This minor change in the guidelines does not seem to support the changes in CMG utilization noted after 2010. [3]

This study has some limitations. First, the study population was restricted to men enrolled in large, employer-provided insurance plans and men over 65 who had an employer-provided retirement benefit after transitioning to Medicare. Thus, this study could not evaluate trends in utilization or cost of UDS in Medicaid enrollees, members of health maintenance organizations (HMOs), or the uninsured. Second, we had no information on the results of the urodynamic tests and only limited information on the actual symptoms that prompted testing, and as a result, we cannot comment on the appropriateness of increasing or decreasing utilization. Third, this analysis did not include information on patient race/ethnicity as those data are not available in this administrative claims database. Finally, the specialty-specific rates presented may underestimate the actual proportion of CMGs performed by urologists since it is possible some urologists are misclassified into other categories.

The strengths of this study include that this is a large, population-based cohort which includes recent data spanning the past decade which has not been captured by earlier research. The sample of CMGs included in this study (153,168) is almost ten times as large as the sample of the next largest study (16,574). [6] As a result of its large sample size, this study’s estimates are precise and granular with detailed information regarding trends by age and across calendar year and region. This study also appropriately restricts the study population to males, preventing the large volume of female UDS from obscuring unique patterns of utilization in men. The use of Poisson models and standardization methods enabled the evaluation of differences across calendar year and region independent of differences in the age of those populations.

This study provides needed information on trends in utilization of UDS over calendar time, indicating that utilization rates, which were on the rise until 2010, began to decline in more recent data years. It is possible that lower utilization rates are due to the CPT coding change which took effect at the beginning of 2010 and subsequent decreases in reimbursement for UDS. [7–10] Despite lower utilization of CMGs in recent years, the proportion of CMGs accompanied by voiding pressure studies, electromyography, and urethral pressure profiles remained constant through 2011 and 2012.

Future research evaluating the usefulness of UDS would have substantial cost implications, given the widespread use of these procedures and relatively high cost (even after decreases in reimbursement). Wide variation in utilization by geographic region may reflect different care practices in different regions of the U.S. Clearer guidelines informing physicians when and how to appropriately use UDS may be needed to reduce practice-variation and is an important area for ongoing study. [17] Future research evaluating utilization of UDS will be needed to determine if the decreasing trend reported in this study continues over the next five or ten years.
